# The relationship between fermentable carbohydrates and post-prandial bowel symptoms in patients with functional bowel disorders

**DOI:** 10.3389/fnut.2023.1060928

**Published:** 2023-02-02

**Authors:** Alice MacIntosh, Phoebe E. Heenan, Morag Wright-McNaughton, Chris Frampton, Paula Skidmore, Catherine L. Wall, Jane Muir, Nicholas Joseph Talley, Nicole Clemence Roy, Richard B. Gearry

**Affiliations:** ^1^Department of Human Nutrition, University of Otago, Dunedin, New Zealand; ^2^Department of Medicine, University of Otago, Christchurch, New Zealand; ^3^High-Value Nutrition National Science Challenge, Liggins Institute at the University of Auckland, Auckland, New Zealand; ^4^Department of Gastroenterology, Monash University, Melbourne, VIC, Australia; ^5^School of Medicine, University of Newcastle, Newcastle, NSW, Australia; ^6^NHMRC Centre of Research Excellence in Digestive Health, University of Newcastle, Newcastle, NSW, Australia; ^7^Riddet Institute, Palmerston North, New Zealand

**Keywords:** FODMAP, functional bowel disorder, fiber, post-prandial bowel symptoms, diet, gastrointestinal symptom, irritable bowel syndrome

## Abstract

**Background and aims:**

A low fermentable oligosaccharide, disaccharide, monosaccharide, and polyols (FODMAP) diet alleviates symptoms of irritable bowel syndrome (IBS). We aimed to investigate the relationship between habitual FODMAP intake and post-prandial bowel symptoms in adults with IBS, functional diarrhoea (FD), or constipation (FD) (functional bowel disorders), and in healthy adults (controls).

**Methods:**

292 participants (173 with functional bowel disorders and 119 controls) completed a food and symptom times diary. Estimated meal portion sizes were entered into the Monash FODMAP Calculator to analyse FODMAP content. Wilcoxon and ANOVA tests were used to investigate the relationship between FODMAP intake and post-prandial bowel symptoms.

**Results:**

IBS participants experienced more post-prandial bowel symptoms compared to participants with other functional bowel disorders or controls. Meals associated with abdominal pain contained on average increased excess fructose (0.31 g vs. 0.18 g, *p* < 0.05), sorbitol (0.27 g vs. 0.10 g, *p* < 0.01), and total FODMAP (3.46 g vs. 2.96 g, *p* < 0.05) compared to meals not associated with pain. Abdominal swelling was associated with increased sorbitol (0.33 g vs. 0.11 g, *p* < 0.01), and total FODMAP (3.26 g vs. 3.02 g, *p* < 0.05) consumption. Abdominal bloating was associated with increased galacto oligosaccharide consumption (0.18 g vs. 0.14 g, *p* < 0.05).

**Conclusion:**

These findings support the role of FODMAP in post-prandial bowel symptom onset, however, the amount and type of FODMAP triggering symptoms vary between individuals. Future research should investigate the relationship between the effect of individual FODMAP consumption on post-prandial bowel symptoms for each subtype, the interaction of FODMAP with differing functional bowel disorders and whether longitudinally symptoms and dietary intake are stable.

## Introduction

Irritable bowel syndrome (IBS) is characterized by recurrent abdominal pain associated with a change in bowel habits (1, 2). Patients with IBS are classified into three subtypes depending on predominant stool form: diarrhea-predominant (IBS-D), constipation-predominant (IBS-C), or mixed phenotype-predominant (IBS-M) (3). Functional diarrhea (FD) and functional constipation (FC) are characterized by a significant change in bowel habits without abdominal pain (4, 5). These are disorders of gut-brain interactions (4), collectively referred to as functional bowel disorders (FBD), and are associated with significant morbidity (2).

While the pathophysiology of FBD is multifactorial, diet plays a significant role in IBS with 63–90% of patients reporting food as a trigger for symptoms (6–8). Reducing consumption of fermentable oligosaccharides, disaccharides, monosaccharides, and polyols (FODMAPs) is effective at improving IBS symptoms in approximately 70% of patients (9–12). FODMAPs include fructose (in excess of glucose, subsequently excess fructose), lactose, fructans, galacto-oligosaccharides (GOS), sorbitol, and mannitol (13). Evidence to date suggests that gut microbiome composition and function of those with IBS may predict response to low FODMAP dietary therapy (14). Whether habitual FODMAP intake shapes the gut microbiome and/or contributes to symptom severity is not fully understood.

The mechanisms by which individual FODMAPs result in bowel symptoms are varied. Some FODMAPs are poorly absorbed (or not absorbed in the case of fructans and GOS) in the small intestine making them available for fermentation by colonic bacteria (15, 16). Some FODMAPs (e.g., fructans) are osmotically active which leads to increased small bowel water and this, in combination with production of gas in the colon *via* microbial fermentation, causes luminal distension (16). Colonic luminal distension caused by consumption of particular FODMAPs coupled with visceral hypersensitivity, dysbiosis and/or altered gastrointestinal (GI) motility may trigger IBS symptoms (15, 17). A study of FBD post-prandial bowel symptom onset found that over 50% of patients’ symptoms worsened within 30 min of ingestion of a meal and 93% within 3 h (18). Anecdotally patients report bowel symptoms after eating but there is a lack of data in free-living populations on the relationship between specific foods and food groups including FODMAPs and post-prandial bowel symptoms in individuals with FBD.

The aim of the present study was to describe the relationship between dietary FODMAP intake and post-prandial bowel symptoms in free-living adults with FBD and healthy adults (controls) and the associations of individual FODMAP with these bowel symptoms.

## Materials and methods

Demographic and dietary data were provided through the Christchurch cOhort to investigate Mechanisms FOr gut Relief and improved Transit (COMFORT) study (19). The COMFORT study was an observational case-control study that recruited FBD participants, and controls. This study was conducted in accordance with the protocol, international conference on Harmonization guidelines, and the ethical principles that have their origin in the Declaration of Helsinki. The COMFORT study protocol was reviewed and approved by the Northern A Ethics Committee (Ref16/NTA/21). Recruited participants were either undergoing colonoscopy or were from the general public in Christchurch, New Zealand. The COMFORT study methodology including recruitment process, biological sample collection process, and cohort description have been published elsewhere (19).

### Participant eligibility

Exclusion criteria for the COMFORT study included blood in the stool, nocturnal symptoms, unexplained weight loss, anemia, pregnancy, a history of bowel disease (e.g., coeliac disease, inflammatory bowel disease) or bowel surgery, or inability to provide informed consent. Participants were diagnosed as IBS, FC, or FD using the most up to date diagnostic criteria for disorders of gut brain interactions (Rome IV) (4). Participants who did not meet the exclusion criteria nor the Rome IV criteria for IBS, FD, or FC were enrolled in the study as controls. Cases and controls who were undergoing endoscopic surveillance for a personal or familial history of sporadic polyps were also recruited.

### Study procedures

#### Food and symptom times (FAST) diaries

COMFORT study participants were asked to complete a FAST diary, a validated dietary assessment tool designed to capture habitual diet and concurrent post-prandial bowel symptoms (20). Prior to colonoscopy, FAST diaries were completed over three consecutive days (20). The last day of the diet diary was at least one day prior to the commencement of bowel preparation for those participants undergoing colonoscopy. The portion size of food or ingredients consumed were estimated by participants using kitchen measurement equipment.

The onset, duration and severity of abdominal pain, swelling/distension, fullness, and bloating onset was recorded during the 3 days of the diet diary. Severity was self-recorded as “Not bad at all, a little bad, somewhat bad, quite bad, or very bad”. This method allowed the capture of multiple bouts of the same symptom over a 24-hour period. The absence or presence, the number and type (using Bristol Stool Chart) of bowel motions was also recorded over the same period. Symptom data from the FAST diaries were transcribed into a Microsoft^®^ Excel^®^ spreadsheet and aligned to the times that meals were ingested. The onset of any symptom (including bowel motions) reported within 3 h after meal ingestion was considered a post-prandial bowel symptom (21).

#### Data entry

Once study data collection was complete, the food diary portion of the FAST diary was entered into two nutrient analysis programmes: Kai-culator© (v1.16a), a dietary assessment software developed by the Department of Human Nutrition at the University of Otago, and The Monash FODMAP Calculator, a FODMAP analysis software created by Monash University, Melbourne, Australia (22).

Kai-culator dietary analysis was completed by experienced researchers and dietitians in the Department of Human Nutrition at the University of Otago. After initial entry to the software, diaries were then checked by two separate experienced researchers to avoid data entry errors. To avoid excluding data unnecessarily the top and bottom 10% of food items were then checked for outliers in protein, carbohydrates and fat which were then checked against the physical diaries in order to ensure data entry errors had not occurred. Data were reported as mean daily intake.

The food diaries were entered into The Monash FODMAP Calculator, a FODMAP analysis software created by Monash University, Melbourne, Australia (22). Food items that were unavailable in the Monash FODMAP Calculator were estimated by creating recipes within the database or entering a substitute with similar FODMAP content or estimated from published FODMAP data (11, 23–27). Recipes for baked items, sauces or meals not included in the Monash FODMAP Calculator, or not provided as a recipe by the participant were taken from the Edmonds Cookery Book© (28). To investigate the impact of FODMAP content on post-prandial bowel symptoms, each eating occasion, whether it was a meal, a single item meal (e.g., an apple) or a snack, was entered into the Monash FODMAP Calculator as a single day and is subsequently referred to as a meal. Mean daily FODMAP intake was also calculated.

The raw macro- and micro-nutrient Kai-culator data and the Monash FODMAP calculator data for each meal consumed was exported into a Microsoft Excel sheet and collated with post-prandial bowel symptom data.

#### Other questionnaires

Participants completed several questionnaires (19) including the Hospital Anxiety and Depression Scale (HADS). Participants with a high HADS Anxiety score (≥11) were omitted from meal and symptom analyses to minimize including participants whose psychological symptoms may be altering the perception of bowel symptoms (29–31).

#### Statistical analyses

All analyses were undertaken using SPSS v25.0. Participants were grouped according to Rome IV diagnostic criteria, and the average daily and meal FODMAP intake for each group was calculated. A one-way ANOVA was performed to determine if any difference in macronutrient or FODMAP consumption existed between Rome IV diagnostic groups and *post-hoc* Games-Howell tests to adjust for multiple comparisons. A two-tailed *p*-value < 0.05 was taken to indicate statistical significance.

Logistic regression analyses were used to generate odds ratios (OR) to compare the proportions of individuals with post-prandial bowel symptoms between Rome IV diagnostic groups. The median number of bowel motions and the Bristol Stool Chart assessment were compared between Rome IV diagnostic groups using Kruskal-Wallis tests, with pairwise comparisons using Mann-Whitney U tests.

For only IBS participants, the number of meals with FAST diary symptoms [abdominal pain, swelling, fullness, bloating, the presence of any of these symptoms (“any symptom”), or bowel motions] recorded within-in 3 h of meal consumption was compared between the dietary fiber (g) and FODMAP content, categorized as low (<0.5 g) or high (≥0.5 g), (31) of meals using Wilcoxon signed-rank non-parametric tests. A two-tailed *p*-value < 0.05 was taken to indicate statistical significance.

## Results

Completed FAST diaries were returned by 292 (93%) participants. Table 1 summarizes the demographic characteristics of participants in these analyses. The mean age of the participants was 53.1 years (range 19 to 70 years); 71.6% were female, and of the total cohort: 40.6% had IBS, 13.3% had FC, and 5.1% had FD.

**TABLE 1:** Demographic characteristics of participants with complete food and symptom times (FAST) diaries.

	FAST diaries completed (% of total cohort) *n* (%)	Age (mean ± SD)	Female *n* (%)
IBS-D	56 (19)	52.3 ± 11.7	43 (77)
IBS-C	27 (9)	50.3 ± 12.1	26 (96)
IBS-M	35 (12)	49.6 ± 12.3	30 (86)
Total IBS	118 (40)	51.0 ± 12.2	99 (84)
FD	15 (5)	57.2 ± 12.3	12 (80)
FC	40 (14)	57.6 ± 12.3	29 (73)
FD + FC	55 (19)	57.5 ± 12.4	41 (75)
Total FBD cases	173 (59)	52.3 ± 10.8	140 (81)
Controls	119 (41)	53.1 ± 12.3	69 (58)
Total	292	53.1 ± 12.3	209 (72)

FAST, food and symptom times; IBS, irritable bowel syndrome; IBS-D, diarrhea predominant IBS; IBS-C, constipation predominant IBS; IBS-M, mixed phenotype predominant IBS; FD, functional diarrhea; FC, functional constipation; FBD, functional bowel disorders; FAST, food and symptom times diary; N, population size; SD, standard error of the mean.

### Dietary intake

There was a significant difference in average daily intake of energy, carbohydrate, and protein between FBD cases and controls (Table 2). FBD cases also had a lower dietary fiber intake (20.2 g vs. 24.0 g, *p* < 0.05). There was no significant difference in average daily intake of total or individual FODMAPs between the Rome IV criteria groups (Table 2).

**TABLE 2:** Mean dietary intake of macronutrients and fermentable oligosaccharide, disaccharide, monosaccharide, and polyols (FODMAPs) between Rome IV diagnostic groups and controls.

	Mean (SD)					
Nutrient (g/day)	IBS-D (*n* = 56)	IBS-M (*n* = 35)	IBS-C (*n* = 27)	FD (*n* = 15)	FC (*n* = 40)	Controls (*n* = 119)
Energy (kJ)	7,378 (303)	7,395 (368)	7,148 (469)	7,547 (690)	7,196 (248)	7,843 (207)
Carbohydrate	175.8 (7.1)	179.6 (11.1)	171.5 (10.8)	175.3 (17.9)	184.9 (7.3)	196.8 (6.2)
Protein	74.3 (3.2)	73.7 (3.0)	73.9 (5.0)	72.4 (6.5)	67.8 (2.2)	78.9 (2.2)
Fat	79.6 (4.6)	77.5 (4.3)	74.1 (6.6)	82.8 (9.4)	72.7 (4.1)	77.5 (2.8)
Fiber	20.3 (1.0)	19.0 (1.1)*	21.6 (2.0)	21.2 (2.8)	19.9 (1.1)	24.0 (1.0)
Total FODMAP with lactose	19.8 (1.7)	21.0 (1.2)	22.9 (3.2)	19.0 (2.7)	23.2 (1.8)	24.2 (2.9)
Total FODMAP without lactose	8.0 (1.0)	7.7 (0.6)	8.2 (0.8)	7.7 (1.0)	8.1 (0.7)	10.3 (1.9)
Excess fructose	1.4 (0.3)	1.2 (0.2)	1.5 (0.2)	1.3 (0.3)	1.6 (0.4)	1.6 (0.2)
Lactose	12.1 (1.4)	13.1 (2.1)	14.3 (2.7)	11.1 (11.1)	15.0 (1.4)	14.2 (1.3)
Sorbitol	1.0 (0.4)	0.7 (0.1)	1.0 (0.2)	0.8 (0.2)	1.1 (0.2)	1.1 (0.1)
Mannitol	0.6 (0.1)	0.6 (0.1)	0.5 (0.1)	0.6 (0.2)	0.5 (0.1)	0.5 (0.1)
Fructans	3.8 (0.2)	4.5 (0.5)	4.5 (0.6)	4.1 (0.4)	4.1 (0.5)	5.7 (1.6)
GOS	1.0 (0.1)	1.0 (0.1)	1.1 (0.2)	1.0 (0.2)	1.0 (0.1)	1.1 (0.1)

FODMAP, fermentable oligosaccharides, disaccharides, monosaccharides, and polyols; GOS, galacto-oligosaccharides; IBS, irritable bowel syndrome; IBS-D, diarrhea predominant IBS; IBS-C, constipation predominant IBS; IBS-M, mixed phenotype predominant IBS; FD, functional diarrhea; FC, functional constipation. *p < 0.05 compared to controls.

### Proportion of participants experiencing post-prandial bowel symptoms associated with meals

**FIGURE 1:**
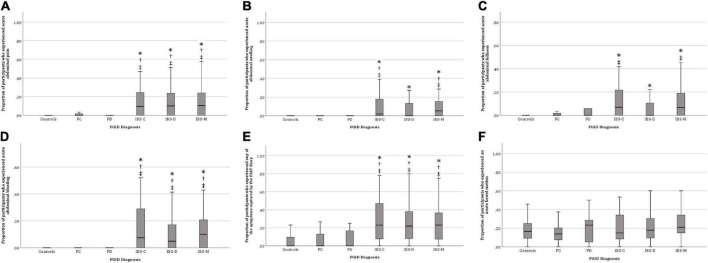
Proportion of participants who experienced post-prandial bowel symptoms. **(A)** Abdominal pain, **(B)** Abdominal swelling, **(C)** Abdominal fullness, **(D)** Abdominal bloating, **(E)** Any gastrointestinal symptom, and **(F)** Bowel motion. IBS, irritable bowel syndrome; IBS-D, diarrhea predominant IBS; IBS-C, constipation predominant IBS; IBS-M, mixed phenotype predominant IBS; FD, functional diarrhea; FC, functional constipation. *p < 0.05 compared with controls, †*p* < 0.05 compared to FC, ‡*p* < 0.05 compared to FD.

Figure 1 shows that IBS participants, regardless of subtypes, were more likely to experience acute abdominal pain, swelling, fullness, bloating, and “any symptoms” associated with meals than controls.

Table 3 summarizes the median proportion and odds of participants in each Rome IV diagnostic group experiencing post-prandial bowel symptoms captured by FAST diaries. Reporting “any symptom” in the FAST diary was significantly associated with IBS-D [OR 4.0 (95% CI 3.3–5.0, *p* < 0.01)], IBS-C [OR 6.9 (95% CI 5.5–8.7, *p* < 0.01)], and IBS-M [OR 4.0 (95% CI 3.1–5.0, *p* < 0.01)] compared to controls. Abdominal pain was significantly associated with IBS-D [OR 7.2 (95% CI 5.0–9.1, *p* < 0.01)], IBS-C [OR 7.5 (95% CI 5.4–10.5, *p* < 0.01)], and IBS-M [OR 6.2 (95% CI 4.5–8.7, *p* < 0.01)] compared to controls. Abdominal swelling was 7.2 times more likely to be reported in IBS-D (95% CI 4.7–10.8, *p* < 0.01), 12.3 times in IBS-C (95% CI 8.1–18.8, *p* < 0.01), and 6.1 times in IBS-M (95% CI 3.9–9.5, *p* < 0.01) participants compared to controls. IBS-D was significantly associated with post-prandial bloating [OR 7.7 (95% CI 5.2–11.5, *p* < 0.01)], as was IBS-C [OR 17.5 (95% CI 11.8–26.1, *p* < 0.01)], and IBS-M [OR 8.9 (95% CI 5.9–13.4, *p* < 0.01)] compared to controls. Additionally, bloating was 3.4 times (95% CI 1.7–6.8, *p* < 0.01) more likely in FD participants compared to controls. Finally, post-prandial fullness was significantly associated with IBS-D [OR 1.7 (95% CI 1.2–2.2, *p* < 0.01)], IBS-C [OR 3.4 (95% CI 2.5–4.5, *p* < 0.01)], and IBS-M [OR 2.2 (95% CI 1.6–2.9, *p* < 0.01)].

**TABLE 3:** Odds of post-prandial bowel symptoms by Rome IV functional bowel disorder diagnostic group.

		IBS-D	IBS-C	IBS-M	FD	FC	Controls
Pain	*N* (%)	111 (9)	79 (17)	68 (15)	0 (0)	44 (6)	47 (2)
	Odds ratio (95% CI)	7.2 (5.0–9.1)**	7.5 (5.4–10.5)**	6.2 (4.5–8.7)**	0.2 (0.0–1.2)	2.2 (1.5–3.3)**	1.0
Swelling	*N* (%)	67 (9)	62 (13)	42 (9)	8 (4)	4 (1)	31 (2)
	Odds ratio (95% CI)	7.2 (4.7–10.8)**	12.3 (8.1–18.8)**	6.1 (3.9–9.5)**	2.9 (1.4–6.2)**	0.4 (0.1–1.1)	1.0
Fullness	*N* (%)	55 (7)	66 (14)	54 (12)	13 (6)	34 (5)	84 (4)
	Odds ratio (95% CI)	1.7 (1.2–2.2)**	3.4 (2.5–4.5)**	2.2 (1.6–2.9)**	1.2 (0.7–2.1)	0.9 (0.6–1.3)	1.0
Bloating	*N* (%)	78 (10)	85 (18)	59 (13)	10 (5)	16 (2)	21 (1)
	Odds ratio (95% CI)	7.7 (5.2–11.5)**	17.5 (11.8–26.1)**	8.9 (5.9–13.4)**	3.4 (1.7–6.8)**	0.3 (0.8–2.6)	1.0
Any symptoms	*N* (%)	187 (24)	159 (13)	124 (26)	25 (12)	70 (10)	128 (7)
	Odds ratio (95% CI)	4.0 (3.3–5.0)**	6.9 (5.5–8.7)**	4.0 (3.1–5.0)**	1.5 (1.0–2.3)	1.2 (0.9–1.6)	1.0
Bowel motion	Median (IQR)	0.2 (0.1, 0.3)	0.2 (0.1, 0.3)	0.2 (0.2, 0.4)	0.2 (0.1, 0.3)	0.1 (0.1, 0.2)	0.2 (0.1, 0.3)
BSC score	Median (IQR)	4.5 (3.8, 5.0)**†‡**	3.8 (3.0, 4.2)	3.7 (3.1, 4.4)	4.1 (4.0, 4.9)**†**	3.0 (2.5, 4.0)	4.0 (3.2, 4.6)**†**

Meal associated with symptoms is expressed as number of meals (N) and percentage of all meals. BSC, Bristol stool chart; IBS, irritable bowel syndrome; IBS-D, diarrhea predominant IBS; IBS-C, constipation predominant IBS; IBS-M, mixed phenotype predominant IBS; FD, functional diarrhea; FC, functional constipation; N, population; CI, confidence interval; IQR, inter quartile range. **p < 0.01 compared to controls. †*p* < 0.05 compared to FC. ‡*p* < 0.05 compared to IBS-C.

There was no significant difference in any of the Rome IV diagnostic categories between-meal consumption and bowel motions (Figure 1). The above results indicate that IBS participants experienced more bowel symptoms associated with meal consumption compared to FC, FD, and control participants. Therefore, subsequent analyses omitted FC, FD, and control participants to avoid type 2 (false negative) statistical error.

### Average dietary fiber and FODMAP intake according to the presence of post-prandial bowel symptoms in IBS participants

Table 4 summarizes the average dietary fiber and FODMAP content of meals associated with bowel symptoms. Meals associated with abdominal pain had significantly higher excess fructose (0.3 g vs. 0.2 g, *p* < 0.05), sorbitol (0.3 g vs. 0.1 g, *p* < 0.01), and total FODMAP (3.5 g vs. 3.0 g, *p* < 0.05) content compared to meals not associated with post-prandial bowel symptoms. Meals with higher sorbitol (0.3 g vs. 0.1 g, *p* < 0.01) and total FODMAP (3.3 g vs. 3.0 g, *p* < 0.05) content were associated with abdominal swelling compared to meals not associated with post-prandial bowel symptoms. Increased fructan (0.8 g vs. 0.6 g, *p* < 0.01), GOS (0.2 g vs. 0.1 g, *p* < 0.01), lactose (2.8 g vs. 1.8 g, *p* < 0.05) and total FODMAP (4.1 g vs. 2.9 g, *p* < 0.01) content was found in meals associated with abdominal fullness compared to meals not associated with post-prandial bowel symptoms. Abdominal bloating was associated with increased meal fiber (3.4 g vs. 2.9 g, *p* < 0.05) and GOS (0.2 g vs. 0.1 g, *p* < 0.05) content. There was no significant difference in aggregated or individual FODMAP meal content associated with post-prandial bowel motions.

**TABLE 4:** Average meal fiber and fermentable oligosaccharide, disaccharide, monosaccharide, and polyols (FODMAP) intake according to the presence of post-prandial bowel symptoms in irritable bowel syndrome (IBS) participants.

		Abdominal pain	Abdominal swelling	Abdominal fullness	Abdominal bloating	Bowel motions
Total fiber (g) (mean)	Symptom present	3.2	3.3	3.4	3.4*	3.2
	Symptom absent	2.9	2.9	2.9	2.9	2.9
Total FODMAP (g) (mean)	Symptom present	3.5*	3.3*	4.1**	3.1	3.2
	Symptom absent	3.0	3.0	2.9	3.0	3.0
Excess fructose (g) (mean)	Symptom present	0.3*	0.3	0.1	0.1	0.2
	Symptom absent	0.2	0.2	0.2	0.2	0.2
Fructan (g) (mean)	Symptom present	0.6	0.6	0.8**	0.6	0.6
	Symptom absent	0.6	0.6	0.6	0.6	0.6
GOS (g) (mean)	Symptom present	0.1	0.2	0.2**	0.2*	0.2
	Symptom absent	0.1	0.1	0.1	0.1	0.1
Lactose (g) (mean)	Symptom present	2.0	1.7	2.8*	2.0	2.0
	Symptom absent	1.9	1.9	1.8	1.9	1.9
Mannitol (g) (mean)	Symptom present	0.1	0.1	0.2	0.1	0.1
	Symptom absent	0.1	0.1	0.2	0.1	0.1
Sorbitol (g) (mean)	Symptom present	0.3**	0.3**	0.1	0.1	0.1
	Symptom absent	0.1	0.1	0.1	0.1	0.1

FODMAP, fermentable oligo-, di-, mono-saccharides and polyols; GOS, galacto-oligosaccharides. *p-value < 0.05. **p-value < 0.01.

### Relationship between increasing FODMAP intake and the proportion of participants experiencing post-prandial bowel symptoms

Significantly more bowel motions occurred following meals containing ≥0.5 g sorbitol. Meals containing ≥0.5 g lactose were associated with decreased frequency of abdominal bloating but increased frequency of bowel motions. Meals containing ≥0.5 g mannitol were associated with decreased frequency of abdominal fullness. A significantly higher number of meals containing ≥0.5 g fructans was associated with abdominal fullness, bloating, and “any symptoms.” Finally, meals containing ≥0.5 g of GOS were associated with decreased frequency of abdominal pain.

## Discussion

In this prospective observational study of free-living FBD cases and controls, we have made a number of novel observations. Firstly, IBS participants are more likely to report functional bowel symptoms after meals than those with FC, FD, or controls. Secondly, IBS participants who experience functional bowel symptoms following meals had a higher intake of total and individual FODMAPs than those without post-prandial bowel symptoms despite no difference in FODMAP consumption between Rome IV diagnostic groups. Finally, there was variability in which FODMAPs lead to symptoms between individuals.

Functional bowel disorders are complex syndromes without a unifying underlying pathophysiology (4). The genesis of symptoms for patients can be driven from a wide range of triggers including diet but also psychological stress and anxiety, medication affects and background risk factors such as a family history of FBDs, previous gastrointestinal infection, physical, or sexual abuse (2, 32, 33). It is also increasingly acknowledged that interactions between the gut microbiota and the host influence the gastrointestinal symptoms (34). This study was unable to adjust for factors that may influence the gut microbiota such as concomitant probiotic supplements use or medication such as proton pump inhibitors. We have shown significant associations between FODMAP intake and post-prandial lower gastrointestinal symptoms in a free-living unselected cohort despite controlling only for those with significant anxiety (HADS > 11).

Higher proportions of meals were associated with abdominal pain, swelling, fullness, bloating, but not bowel motions in IBS participants, compared to controls, FD, and FC participants. As already mentioned, the osmotic actions and fermentation of FODMAPs cause luminal distension (35). Total meal FODMAP content is thought to be a contributing factor of symptom induction (15) and results from the FAST questionnaire support this observation. Meals associated with post-prandial bowel symptoms had an increased total FODMAP content compared to meals not associated with these symptoms. Post-prandial bowel motions were not associated with meal ingestion which was also consistent with another study (36). However, the FAST diary data also showed that the increased content of individual FODMAPs in meals was also associated with post-prandial bowel symptoms in IBS participants.

Increased average intake of excess fructose and sorbitol consumption was found in meals associated with abdominal pain and swelling in IBS participants. The co-ingestion of fructose and sorbitol has been linked to increased colonic bacterial fermentation, although peak gas production associated with bacterial fermentation has not been correlated to IBS symptom onset (27, 37) suggesting an alternative mechanism may be responsible for symptom onset. Similarly, while GOS consumption was associated with abdominal fullness and bloating, a study measuring hydrogen production after GOS consumption was unable to find a difference between GOS consumption or placebo (37). This finding suggests that symptom onset associated with GOS consumption may be independent of bacterial fermentation. However, a dose-dependent association of GOS and bowel symptoms remains relatively understudied (35).

Increased lactose and fructan content were associated with a higher proportion of post-prandial abdominal fullness in IBS participants. High lactose-containing foods are not generally restricted in a low FODMAP diet unless a patient has demonstrated symptoms of lactose intolerance or malabsorption (15, 38). Foods high in fructans and dairy containing products have been associated with satiety and fullness in other FBD cohorts (39, 40). Recent exploratory work suggests that foods rich in fructans and lactose may promote symptoms through the activation of immunoglobulin E^+^ mast cells in close proximity to nerve fibers (41). No data on reported food intolerances were collected, nor were malabsorption or immunoglobulin tests conducted in the COMFORT study.

The COMFORT study participants consumed an average of 22.50 g total FODMAP per day, more than the average daily FODMAP intake in an Australian IBS population (16.3 g) and similar to an elderly New Zealand population (21.7 g) (42, 43). However, similar to other studies, lactose contributed the most to overall FODMAP intake (42, 43). Habitual FODMAP intake and the association with bowel symptom onset remains relatively understudied. Previous studies have assessed dietary intake using food frequency questionnaires (FFQ) and have not attempted to correlate FODMAP intake with bowel symptoms in a three-hour post-prandial window (44, 45). FFQ are inexpensive and easy to complete, however, they have several disadvantages compared to diet diaries (46) including significant recall bias. Furthermore, there is limited ability to convert FFQ data into food composition databases (47). Finally, diet diaries generally correlate more closely with biomarkers of food consumption than FFQ (48) and can more accurately capture post-prandial effects of food. FAST diaries utilize diet diaries and concurrently capture bowel symptoms, allowing for correlations between meal or food intake and symptom onset (20).

Dose-dependent relationships of individual FODMAP and worsening bowel symptoms has been previously demonstrated (37, 49). The COMFORT cohort results were equivocal. Fructans, in particular, appeared to have a dose-dependent relationship with FAST symptoms. However, these results were inconsistent with other FODMAP categories. These results demonstrate the importance of assessing FODMAP sensitivity on an individual basis.

### Strengths

This study had a number of strengths. Firstly, the diagnosis of specific FBDs was made using the most up to date diagnostic criteria for disorders of gut brain interactions (Rome IV). Furthermore, most participants underwent a colonoscopy in the days after their inclusion in the study, reducing the risk of misclassification. Weighed food diaries were used to improve the accuracy of food intake and validated dietary calculators for general and FODMAP diet intake were used to accurately measure the intake of macronutrients, fiber and FODMAPs. Finally, the FAST diary, a validated tool to assess the association between dietary intake and gastrointestinal symptoms was used to capture dietary and patient reported data.

### Limitations

Limitations of this study include challenges common to all studies of dietary intake. An estimated diet diary is more accurate than FFQ but is still subject to a degree of recall bias (8). Additionally, the COMFORT study was observational and cross-sectional. As a result, causal relationships between food ingestion and post-prandial bowel symptoms could not be determined. Associations between other known causes of post-prandial bowel symptoms, such as fruit juices and carbonated beverages, could also not be investigated due to limited food groupings in the food composition database. Similarly, no inference could be made as to whether there was a degree of reverse causality when participants who are experiencing post-prandial bowel symptoms instigated a change in their diets rather than a change in diet causing symptom onset. Furthermore, tests of FODMAP sensitivities were not undertaken. As FODMAP sensitivities are likely highly individualized, the inability to discern individual FODMAP sensitivities may have contributed to the lack of significant associations between ≥0.5 g FODMAP ingestion per meal and the proportion of participants who experienced a symptom(s). A gender effect could not be analyzed due to fewer males in the FBD subgroups and the higher proportion of males in the control group. The findings may not be generalizable to men with post-prandial bowel symptoms.

## Conclusion

In this cross-sectional study of free-living adults with FBD and healthy participants (controls), a higher intake of FODMAPs was associated with post-prandial bowel symptoms. However, the type and amount of FODMAP associated with these symptoms varied between individuals and future research is needed to better understand this relationship. Furthermore, it is not yet known whether associations between post-prandial bowel symptoms and dietary intake are stable longitudinally. This study shows the complexity of the role FODMAPs may play in post-prandial bowel symptoms and highlights the need for individualized management of IBS symptoms by an experienced dietitian.

## Data availability statement

The raw data supporting the conclusions of this article will be made available by the authors, without undue reservation.

## Ethics statement

The studies involving human participants were reviewed and approved by Northern A Ethics Committee, New Zealand (Ref16/NTA/21). The patients/participants provided their written informed consent to participate in this study.

## Author contributions

RG, NR, NT, and CF conceived and designed the study and analyses. RG obtained ethical approval. PH and MW-M collected the data. CF, PH, AM, and MW-M performed the statistical analysis. PH, AM, and CW wrote the manuscript. RG, JM, PS, NT, NR, CW, and CF gave critical feedback on the manuscript. All authors have approved the final manuscript.
